# Mathematical Analysis of Copy Number Variation in a DNA Sample Using Digital PCR on a Nanofluidic Device

**DOI:** 10.1371/journal.pone.0002876

**Published:** 2008-08-06

**Authors:** Simant Dube, Jian Qin, Ramesh Ramakrishnan

**Affiliations:** Fluidigm Corporation, South San Francisco, California, United States of America; National Cancer Institute at Frederick, United States of America

## Abstract

Copy Number Variations (CNVs) of regions of the human genome have been associated with multiple diseases. We present an algorithm which is mathematically sound and computationally efficient to accurately analyze CNV in a DNA sample utilizing a nanofluidic device, known as the digital array. This numerical algorithm is utilized to compute copy number variation and the associated statistical confidence interval and is based on results from probability theory and statistics. We also provide formulas which can be used as close approximations.

## Introduction

### Digital PCR and Digital Array

**Figure 1:**
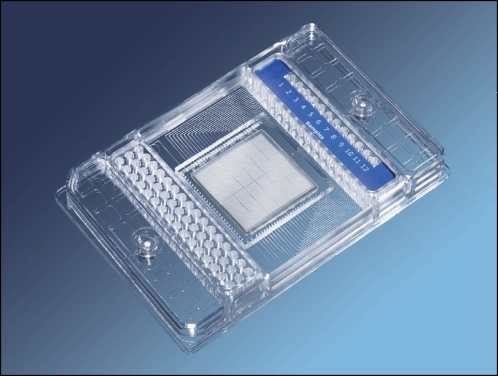
A digital array has 12 panels of 765 reaction chambers each. PCR mixes are loaded into each panel and single DNA molecules are randomly partitioned into the chambers. The digital array can be thermocycled, imaged on a BioMark instrument, and the data analyzed using the Digital PCR Analysis software.

**Figure 2:**
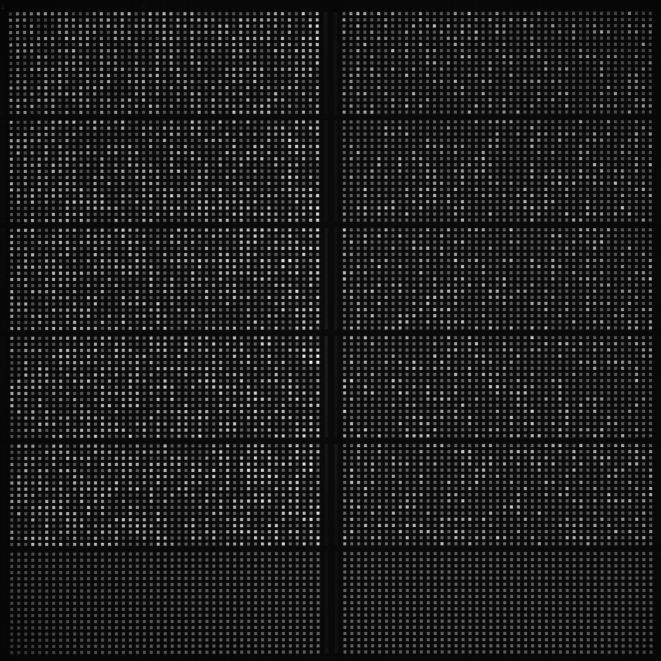
Human genomic DNA NA10860 (left 5 panels) and the RPP30 synthetic construct (right 5 panels) were quantitated using the RPP30 (FAM) assay on this digital array. The two bottom panels are NTC (no template control). Digital PCR Analysis software can count the number of positive chambers in each panel. When two assays with two fluorescent dyes are used in a multiplex digital PCR reaction, two genes can be independently quantitated. This is the basis of the CNV study using the digital array.

Digital PCR conventionally utilizes sequential limiting dilutions of target DNA, followed by amplification using the polymerase chain reaction (PCR) [1], [2]. As a result, it is possible to quantitate single DNA target molecules. We utilize the digital array, which is a novel nanofluidic biochip [2], [3] where digital PCR reactions can be performed (Figure 1) by partitioning DNA molecules, instead of diluting them. This chip utilizes integrated channels and valves that partition mixtures of sample and reagents into 765 nanolitre volume reaction chambers. DNA molecules in each mixture are randomly partitioned into the 765 chambers of each panel (the total volume of the PCR mix in each panel: 6 nl×765 = 4.59 µl). The chip is then thermocycled and imaged on Fluidigm's BioMark real-time PCR system and the positive chambers that originally contained 1 or more molecules can be counted by the digital array analysis software (Figure 2).

### Copy number variation

Copy number variations (CNVs) are the gains or losses of genomic regions which range from 500 bases on upwards in size. Whole genome studies have revealed the presence of large numbers of CNV regions in human and a broad range of genetic diversity among the general population [4], [5], [6]. CNVs have been the focus of many recent studies because of their roles in human genetic disorders [7], [8], [9].

Current whole-genome scanning technologies use array-based platforms (array-CGH and high-density SNP microarrays) to study CNVs. They are high throughput but lack resolution and sensitivity. Real-time PCR is a sequence-specific technique which is easy to perform, but is limited in its discriminating power beyond a 2-fold difference [11], [12].

CNV determination on the digital array is based upon its ability to partition DNA sequences. Given the number of molecules per panel and the dilution factor, the concentration of the target sequence in a DNA sample can be accurately calculated. In a multiplex PCR reaction with 2 or more assays, multiple genes can be quantitated simultaneously and independently, effectively eliminating any pipetting errors if separate reactions have to be set up for different genes. When a single copy reference gene (RNase P in this study, [10]) is used in the reaction, the ratio of the target gene to the reference gene would reflect the copy number per haploid genome of the target gene.

### Primary contribution of this paper

In this paper we will show that the digital array provides a robust and easy-to-use platform to study CNVs. We have derived a mathematical framework to calculate the true concentration of molecules from the observed positive reactions in a panel. We also show how to perform statistical analysis to find the 95% confidence intervals of the true concentrations and the ratio of two concentrations in a CNV experiment using the digital array with multiplex PCR.

The copy number variation problem can be stated as follows. *Given two counts h*_1_ *and h*_2_ *of positive chambers for two genes in a digital array panel, how can one estimate a ratio of true concentrations r = λ*_1_ */λ*_2_ *of the two genes and a confidence interval* [*r_Low_*, *r_High_*] *on the estimation?*

Our approach is built on well-known tools and techniques from statistics. It decomposes the problem into two parts.

1. Given a count *h* of positive chambers, how can one estimate the true concentration *λ* of target molecules in the DNA sample and a confidence interval [*λ_Low_*, *λ_High_*] on this estimation?
2. Given estimated true concentrations *λ*_1_ and *λ*_2_ of the reference gene and the target gene, respectively, in the DNA sample and their respective confidence intervals, how can one estimate the ratio *r* = *λ*_1_/*λ*_2_ and a confidence interval [*r_Low_*, *r_High_*] on this estimation?

It turns out that the first question can be answered by applying sampling and estimation theories from statistics and probability, and the second question can be answered by a numerical algorithm based on generalization of a mathematical theorem.

For related work on answering the first question, using Bayesian approach, see unpublished preprint by Warren et al. titled “The Digital Array Response Curve” dated March 2007 at http://thebigone.stanford.edu/papers.htm. Warren et al. assumed a uniform probability distribution of number of molecules, with maximum number assumed to be 4000, and using Bayesian and combinatorial methods, presented a solution. The confidence interval obtained using Bayesian probabilistic framework, is often referred to as *credible interval or Bayesian confidence interval* which requires one to incorporate problem-specific contextual information from the prior distribution.

This paper differs from this prior work by Warren et al. in two different ways. First, we consider the parameter *λ* to be a fixed constant, unlike having a probability distribution as in Bayesian approach. Second, in addition to providing the answer to the first question, we are interested in estimating the confidence interval of the ratio of two concentrations which is new work. For difference between credible interval and confidence interval, see [13]. Both approaches give good results depending upon the question one is trying to answer.

We will prove mathematical correctness of our results in this paper and present simulation results to help the reader build useful insight. Finally, we present actual CNV experiments on the digital array with known ratios and show the results using the techniques developed in this paper.

## Results

DNA quantitation in the digital array is based on the partitioning of a PCR reaction into an array of several hundreds or even few thousands of chambers or wells. One panel of the digital array consists of 765 chambers and one can use up to 12 panels at a time. If the concentration of the target molecules is low in the DNA sample, most of the chambers capture either one or no molecules and the number of positive chambers at the end point of the PCR yields close approximation to the true concentration of the target. However, if the number of molecules is large, then there is greater probability of several molecules being in the same chamber, and therefore the number of positive chambers would be significantly lower compared with the number of molecules in the chambers.

We are interested in estimating the true concentration of the molecules in the DNA sample from which we extracted 6 nl×765 = 4.59 µl of sample for each panel.

Consider the universe of infinite number of the digital array chambers filled with an infinite amount of the DNA sample where the true concentration of the target molecules is *λ* per chamber (per 6 nl). The true concentration is an unknown population parameter of this infinite DNA sample. If a chamber gets no molecule then it constitutes failure in the sense of Bernoulli experiment. If it gets one or more molecules, that is, if it gets a “hit” and is therefore positive, then it constitutes success. Let the probability of success be *p*. Note that *p* is an unknown population parameter. We will use the standard hat notation to denote sample estimators of population parameters. For example, *pˆ* and 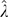 will denote the estimators of *p* and *λ*, respectively.

### Relationship between *p* and *λ*

One can model *K*, the number of molecules in each chamber as a Poisson process, and this gives the relationship between *p* and *λ* as follows
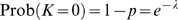
Alternatively, consider *M* molecules randomly distributed in *C* chambers. The probability of any given molecule being in any given chamber is 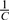. So the probability *p* of a given chamber having at least one molecule is
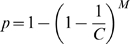
Since *M* = *λC*, we have
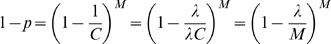
As number of chambers becomes arbitrarily large, the above approaches *e* ^−*λ*^. Therefore,
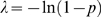
which establishes the relationship between *λ* and *p*.

### Confidence Intervals for estimation of *p* and *λ*

**Figure 3:**
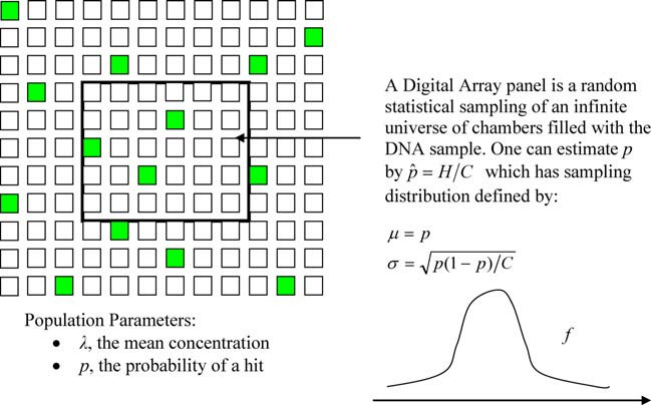
Consider an infinite universe of chambers. A digital array panel is a finite sampling of this universe. The goal is to determine *λ*, the mean number of the target molecules per chamber in the DNA sample. The number of positive chambers, which have hits of one or more molecules, shown as filled green squares in the panel with *C*( = 765) chambers is *H*.

A chamber getting a hit or no hit is a binomial process, same as toss of a coin, with success probability *p*. Let the number of positive chambers in the panel be *H*. Consider 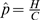 as an estimator of *p*. It is well known that *pˆ* is an unbiased estimator of *p* and has expectation *p* and standard deviation 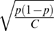 and its sampling distribution *f*(*pˆ*) is approximately normal for large *C*. See Figure 3 for illustration of the above ideas.

See [13], [14], [15] for extensive literature on obtaining confidence interval for the estimation of binomial probability. It is referred to as binomial sign test when the test statistic can be approximated with the chi-square distribution, specifically through the use of the chi-square goodness-of-fit. An alternative and equivalent approximation is obtained by using the normal distribution and then the test is referred to as the population proportion test, see [15].

If *C* is large enough, then the confidence limits are approximately given by
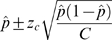
For 95% confidence interval, *z_c_* = 1.96. For the digital array, *C* is an integral multiple of 765 and is comfortably large enough for the above approximation.

**Figure 4:**
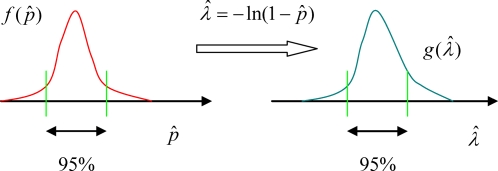
From the sampling distribution of estimation of p, one can obtain the sampling distribution of estimation of λ.

Define the estimator of *λ* as
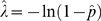
Since the probabilities in any given differential area of a probability density function are preserved under change of variables, the 95% confidence interval [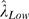, 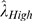] is directly given as follows 
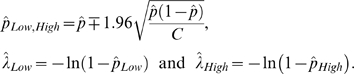
See Figure 4 for illustration.

### Expectation of estimation of *λ*

Let a random variable *X* have probability density function *f_X_*(*x*). If *h*(*x*) is either increasing or decreasing in *x*, then *U* = *h*(*X*) has density function given by
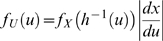
which follows from the fact that probabilities in any given differential area have to be invariant under change of variables, see [16]. Furthermore,
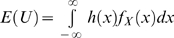
which can be expanded using Taylor series expansion of *h*(*x*) around the mean *ξ* = *E*(*X*), as follows
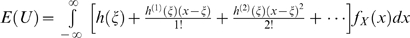
Since in our case, we have the following
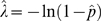
therefore, in above, we have *x* = *pˆ*, 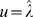 and *h*(*x*) = −ln(1−*x*). Since 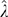 is a monotonically increasing function of *pˆ*, one can get the sampling distribution of 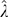 from the sampling distribution of *pˆ* as
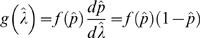
Note that due to nonlinear relationship between 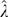 and *pˆ*, one can not make assumptions about *g*. In general, *g* is not normal and 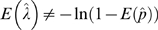.

Now we derive an approximation for 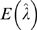 from the Taylor series expansion shown above. Higher order central moments of Gaussian function *f*(*pˆ*) with mean *p* are
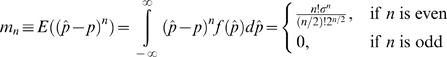
For proof see [17]. Since *f*(*pˆ*) has very small *σ* due to very large number of chambers, the higher order terms for all *n*>0 in the Taylor expansion are small,
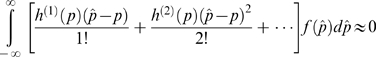
and therefore the only contributing term is when *n* = 0, which implies
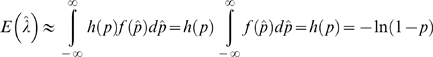
As *C*→∞, 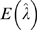 approaches the true concentration *λ* of molecules.

### Simulation Results on Estimation of *p*

It is informative and useful to run a simulation experiment on the computer to see how the real-world matches with the theory developed above. For this purpose, one can use a random number generator and a computer program to simulate the universe of the digital array chambers.

If a panel has *C* chambers, consider a universe of *C*×*K* many chambers where *K* is a large number chosen for simulation. Choose some value of *λ* as the true concentration of molecules in one chamber. Therefore, in total, there will be *λ*×*C*×*K* molecules. Assign each of these molecules randomly to one of the chambers.

Extract *K* panels out of this universe and for each of the panels, compute 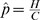 as an estimator of *p* and plot its histogram over all the *K* panels. The mean should be *p* = 1−*e* ^−*λ*^ and standard deviation should be 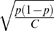. For each of these panels, estimate *λ* and compute the 95% confidence interval. In 95% of the *K* panels, the true value of *λ* should lie within the confidence interval.

**Figure 5:**
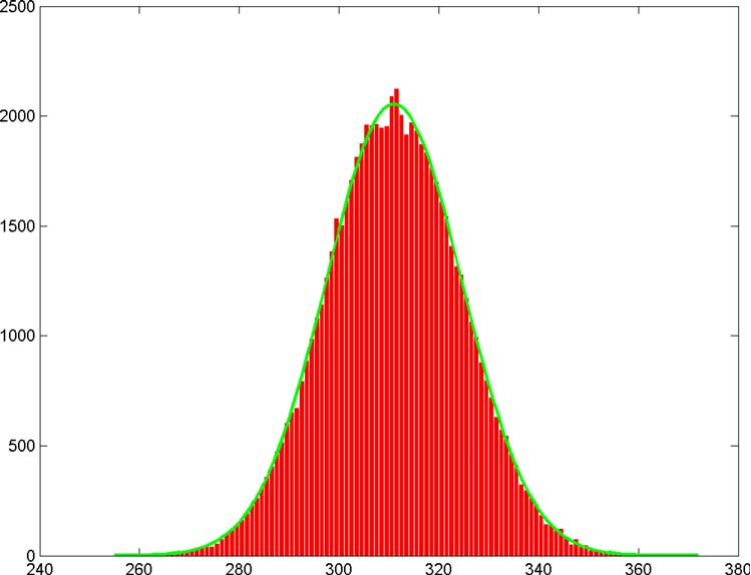
Histogram of number of positive chambers *H* = *P*×*C* obtained by choosing *M* = 400 as the mean number of molecules per panel over 70 thousand panels and running a simulation using a random number generator. The green curve is the sampling distribution predicted by the theory.

For our simulation experiments we chose *M* = 400, that is, 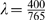. We chose *K* = 70000. In Figure 5 we show the histogram of *H* which is really same as distribution of *P* scaled by a factor of 765. In Table 1 we show how the predicted values match with the actual simulation values. In the same way, the sampling distribution of number of molecules matched with what is predicted by theory. Though the results of the simulation follow from elementary probability, we conducted these simulations in order to build more advanced simulations for ratios of concentrations later. They also illustrate the meaning of the confidence interval.

**Table 1:** Comparison of the metrics of histogram, shown in Figure 5, of number of positive chambers obtained in simulation with those predicted by the theory.

	Theoretical Predictions	Simulation Results
Mean	311.5	311.48
Standard Deviation	13.59	13.58
Percent of times *M* = 400 lies in the computed 95% confidence interval	95%	94.44%

### Determination of Ratio of Concentrations

In previous section, we established a method for estimating the true concentration of the target molecules in the DNA sample from the count of positive chambers as well as the 95% confidence interval for this estimation. We also showed how the sampling distribution 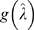 is related to the sampling distribution *f*(*pˆ*).

In CNV, the goal is to determine ratio of true concentrations of two genes, one being reference gene and the other being test gene, and associated confidence interval, which we now accomplish in next subsections.

### Fieller's Theorem and its geometric interpretation

Let the sampling distributions of the test gene and the reference gene be 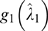 and 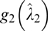, respectively. If these distributions were normal, then one can make use of Fieller's Theorem [18], [19].

**Figure 6:**
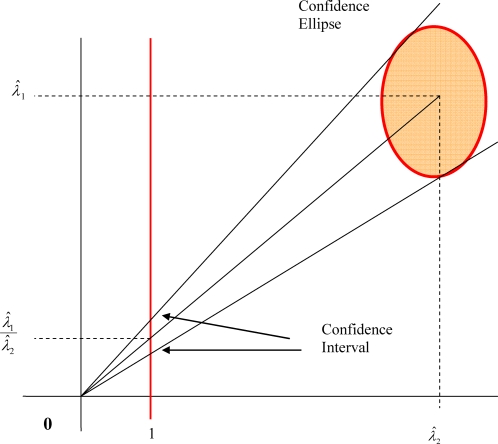
Geometric interpretation of Fieller's Theorem to compute confidence interval of ratio of two normally distributed random variables 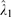 and 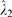 in which confidence ellipse of the joint sampling distribution is projected on a vertical line.

However, as mentioned in previous section, one can not make this assumption in general. It is useful to go through the geometric interpretation of Fieller's theorem so that one can solve the problem for arbitrary sampling distributions. See Figure 6 for geometric interpretation of Fieller's Theorem [20], [21].

Assume 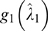 and 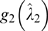 are normal. For 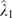 and 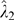, the ratio 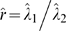 can be shown as the slope of the line in the two-dimensional plane which passes through the origin and the 2-D point (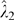, 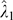). Luxburg et al. show in [20], [21] how a confidence ellipse in the two-dimensional plane can be constructed. Consider the two lines which pass through the origin and are tangents to this ellipse. The intersection of these lines with the vertical line at 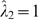 gives the desired confidence interval.

### Ratio of concentrations

In this paper we have presented data in a controlled experimental system, where a synthetic DNA construct was spiked into human cell line DNA at different concentrations. In this case, the synthetic construct, which was to the RPP30 gene, was used as the target, and the RNase P gene which was endogenous to the human cell line, was used as the reference gene. The two genes were identified using two separate PCR reactions, using separate PCR primers and probes. Since there is no reason to assume that the amplification and detection of the target and reference genes are linked, 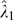 and 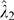 are independent variables.

It is easy to see from the proof of Fieller's theorem and its geometric interpretation that one can compute sampling distribution *q* of the ratio estimator 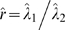 as follows:
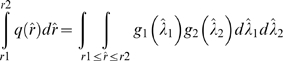
This can be interpreted as cutting out thin wedges in the joint distribution of 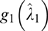 and 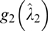 and accumulating the probabilities inside the wedge to compute the function *q* in the corresponding thin interval of the ratio. This is the basis of our numerical algorithm which implements integration in order to compute *q*(*rˆ*):

1. Build histograms of sampling distributions 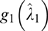 and 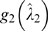. The tails of the histograms where probabilities become very small are approximated by zero.
2. Build a histogram of sampling distribution *q*(*rˆ*) of 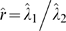 by considering each bin [*r*_1_, *r*_2_] and by adding all the joint probabilities of different values of concentrations which give a ratio *rˆ*∈[*r*_1_, *r*_2_].
3. Compute the mean and the 95% confidence interval from the ratio histogram.

**Figure 7:**
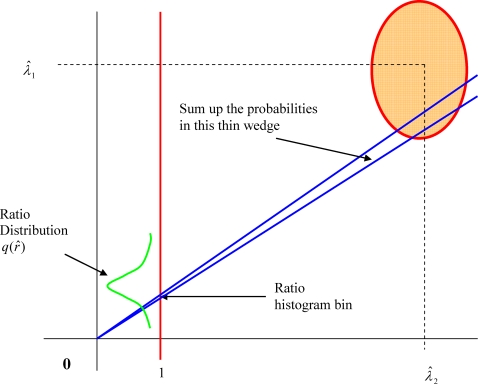
Illustration of a numerical projection algorithm to compute the sampling distribution of ratio of two random variables with arbitrary probability distributions by slicing the 2-D space into thin wedges and accumulating the joint probabilities in the wedges. Most of the contribution would come from the confidence ellipse region.

See Figure 7 for illustration of the above algorithm.

One can still use direct formulas, as an approximation, to compute confidence interval as follows.

The means of 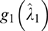 and 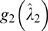 are *λ*_1_ and *λ*_2_ respectively. Let the standard deviations be *σ_x_* and *σ_y_* respectively. For given estimations 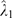 and 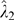, assuming that distributions are normal, it follows from the analysis in [20], [21] that the boundary of the confidence ellipse for a given confidence level *z_c_* would be defined by
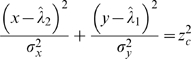
It is easy to generalize this to the case, under a reasonably close approximation, when we have asymmetric distributions which are assumed to be normal in each of the four quadrants of the coordinate system centered at (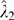, 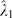). Then the confidence region is made of union of four quadrant-wise elliptic regions.

Let the asymmetric confidence intervals for specified *z_c_* and the two concentrations be [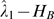, 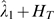] and [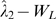, 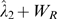].

If *W_R_* = *W_L_* = *z_c_σ_x_* and *H_T_* = *H_B_* = *z_c_σ_y_*, it is symmetric case [20], [21]. Using simple algebraic manipulations, it can be shown, as in symmetric case, that the slopes of lines that will be tangents to this union of four quadrant-wise ellipses will be
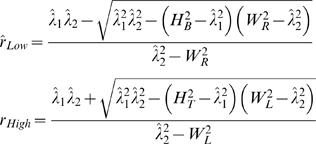
The above equations can be used as an approximation though numerical algorithm will give more accurate results as the algorithm does not make any assumptions and works with arbitrary sampling distributions.

One detail has to be mentioned. Special care has to be taken if the confidence region gets too close to 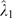 axis when 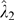 is small. If it touches 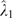 axis, then *r_High_* = ∞. If either 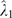 or 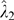 is too small, one can build respective histogram with smaller bin size to get more accurate results.

See Table 2 for summary of equations derived in order to solve the copy number variation problem. Though the numerical approach based on histograms is recommended as it does not make assumptions, these direct formulas can be used as close approximation.

**Table 2:** Given number of chambers *C* and counts *H*_1_ and *H*_2_ of the positive chambers in a digital array for the target gene and the reference gene, respectively, list of formulas needed to analyze copy number variation.

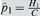 , 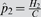
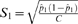 , 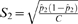
*pˆ*_1,*Low*_ = *pˆ*_1_−1.96*S*_1_, *pˆ*_1,*High*_ = *pˆ*_1_+1.96*S*_1_
*pˆ*_2,*Low*_ = *pˆ*_2_−1.96*S*_2_, *pˆ*_2,*High*_ = *pˆ*_2_+1.96*S*_2_
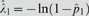 , 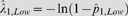 , 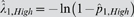
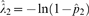 , 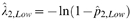 , 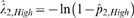
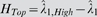 , 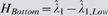
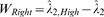 , 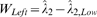
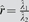
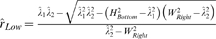
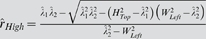

See the details in the paper for assumptions made so that these equations are close approximations to actual values.

### Simulation Results on Estimation of Ratio

We conducted simulation studies, using a random number generator and a computer program as in previous section, by choosing a ratio of 2 of concentrations of two genes, which are independent of each other, and building a distribution of estimated ratios over 50 thousand panels. In 94.9% of the panels, the true chosen ratio did lie in the computed confidence intervals thereby showing the correctness of our mathematical analysis.

### Actual Results on Estimation of Ratio

**Figure 8:**
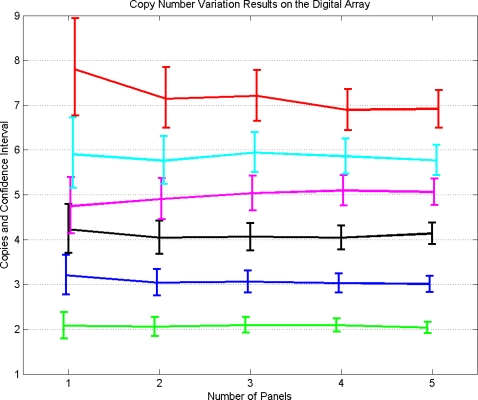
Results of actual CNV experiments on the digital array with varying number of copies of the target gene. In total, 6 different known ratios were estimated by running the experiments for varying number of panels. The graphs for different numbers of copies are slightly staggered to allow visual comparison of overlap of the 95% confidence intervals.

The copy number variation results for known ratios of 1, 1.5, 2, 2.5, 3 and 3.5 are shown in Figure 8. Materials and methods for this experiment are discussed in next section. As the number of panels *P* increases, then the number of chambers *C* = 765 *P*increases and therefore the estimation of the ratio becomes more accurate as well as the confidence interval shrinks. When only 1 panel is used, there is significant overlap between 95% confidence intervals of certain ratios e.g. between ratio 2 and 2.5. There is no overlap when 3 or more panels are used. In all cases the known ratio lies within the computed 95% confidence interval. Note that using mathematical analysis one can find optimal numbers of positive chambers for each ratio which give smallest confidence intervals and which will therefore improve the results.

In summary, Fluidigm's digital array is capable of accurately quantitating DNA samples and is a valuable platform for studying copy number variation. It is a robust technology that is sequence-specific, easy-to-use, and extremely flexible. We have presented mathematical and algorithmic solutions to analyze CNV on a digital array. The solution is an elegant application of statistical sampling and estimation theories to such an important real-world problem. We have shown how one can compute the true concentration of a target sequence in a DNA sample and the associated confidence interval on this estimation, and how one can compute the ratio of true concentrations of multiple sequences and the associated confidence interval on the estimation of this ratio.

## Materials and Methods

A 10-µl reaction mix is normally prepared for each panel. It contains 1× TaqMan Universal master mix (Applied Biosystems, Foster City, CA), 1× RNase P-VIC TaqMan assay, 1× TaqMan assay for the target gene (900 nM primers and 200 nM FAM-labeled probe), 1× sample loading reagent (Fluidigm, South San Francisco, CA) and DNA with about 1,100–1,300 copies of the RNase P gene. 4.59 µl of the 10-µl reaction mix was uniformly partitioned into the 765 reaction chambers of each panel and the digital array was thermocycled on the BioMark system. Thermocycling conditions included a 95°C, 10 minute hot start followed by 40 cycles of two-step PCR: 15 seconds at 95°C for denaturing and 1 minute at 60°C for annealing and extension. Molecules of the two genes were independently amplified. FAM and VIC signals of all chambers were recorded at the end of each PCR cycle. After the reaction was completed, Digital PCR Analysis software (Fluidigm, South San Francisco, CA) was used to process the data and count the numbers of both FAM-positive chambers (target gene) and VIC-positive chambers (RNase P) in each panel.

A spike-in experiment was performed using a synthetic construct to explore the digital array's feasibility as a robust platform for the CNV study. A 65-base oligonucleotide was ordered from Integrated DNA Technologies (Coralville, IA) that is identical to a fragment of the human RPP30 gene. The sequences of the primers and FAM-BHQ probe used to amplify this construct are from Emery et al [22]. The primers and probe were ordered from Biosearch Technologies (Novato, CA).

Both RPP30 synthetic construct and human genomic DNA NA10860 (Coriell Cell Repositories Camden, NJ) were quantitated using the RPP30 assay on a digital array. Different amounts of RPP30 synthetic construct was then added into the genomic DNA so that mixtures with ratios of RPP30 to RNase P of 1∶1 (no spike-in), 1∶1.5, 1∶2, 1∶2.5, 1∶3, and 1∶3.5 were made simulating DNA samples containing 2 to 7 copies of the RPP30 gene per diploid cell.

These DNA mixtures were analyzed on the digital arrays as described above. Five panels were used for each mixture and 400–500 RNase P molecules were present in each panel. The ratios of RPP30/RNase P of all samples were calculated using the techniques developed in this paper. For each ratio, we did pooled analysis by adding the numbers of positive chambers in the first *P* = 1,2,3,4,5 panels. The results are summarized in the previous section and in Figure 8.

## References

[1] Vogelstein B, Kinzler KW (1999). Digital PCR. Proc. Natl. Acad.. Sci U S A.

[2] Spurgeon SL, Jones RC, Ramakrishnan R (2008). High Throughput Gene Expression Measurement with Real Time PCR in a Microfluidic Dynamic Array.. PLoS ONE.

[3] Sindelka R, Jonak J, Hands R, Bustin SA, Kubista M (2007). Intracellular expression profiles measured by real-time PCR tomography in the Xenopus laevis oocyte.. Nucleic Acids Research.

[4] Iafrate AJ, Feuk L, Rivera MN, Listewnik ML, Donahoe PK (2004). Detection of large-scale variation in the human genome.. Nat Genet.

[5] Sebat J, Lakshmi B, Troge J, Alexander J, Young J (2004). Large-scale copy number polymorphism in the human genome.. Science.

[6] Redon R, Ishikawa S, Fitch KR, Feuk L, Perry GH (2006). Global variation in copy number in the human genome.. Nature.

[7] Wong KK, deLeeuw RJ, Dosanjh NS, Kimm LR, Cheng Z (2007). A comprehensive analysis of common copy-number variations in the human genome.. Am J Hum Genet.

[8] Ropers HH (2007). New perspectives for the elucidation of genetic disorders.. Am J Hum Genet.

[9] Lupski JR (2007). Genomic rearrangements and sporadic disease.. Nat Genet.

[10] Baer M, Nilsen TW, Costigan C, Altman S (1990). Structure and transcription of a human gene for H1 RNA, the RNA component of human RNase P.. Nucleic Acids Res.

[11] Carter NP (2007). Methods and strategies for analyzing copy number variation using DNA microarrays.. Nat Genet.

[12] Lo YM, Lun FM, Chan KC, Tsui NB, Chong KC (2007). Digital PCR for the molecular detection of fetal chromosomal aneuploidy.. Proc Natl Acad Sci USA.

[13] Kapadia AS, Chan W, Moyé LA (2005). Mathematical Statistics with Applications.

[14] Motulsky H (1995). Intuitive Biostatistics.

[15] Sheskin DJ (2000). Handbook of Parametric and Nonparametric Statistical Procedures.

[16] Scheaffer RL (1990). Introduction to Probability and its Applications..

[17] Papoulis A (1965). Probability, Random Variables, and Stochastic Processes.

[18] Fieller E (1932). The distribution of the index in a normal bivariate population.. Biometrika.

[19] Fieller E (1954). Some problems in interval estimation.. J Roy Statist Soc Ser: B.

[20] von Luxburg U, Franz VH (2002). Confidence Sets for Ratios: A Purely Geometric Approach To Fieller's Theorem..

[21] von Luxburg U, Franz VH (2007). A Geometric Approach to Confidence Sets for Ratios: Fieller's Theorem, Generalizations, and Boostrap.. http://arxiv.org/abs/0711.0198.

[22] Emery SL, Erdman DD, Bowen MD, Newton BR, Winchell JM (2004). Real-time reverse transcription-polymerase chain reaction assay for SARS-associated coronavirus.. Emerg Infect Dis.

